# Giant intracranial *Brucella* abscess after head trauma: a Case Report of neurobrucellosis in an urban elderly male without exposure history

**DOI:** 10.3389/fmed.2025.1676548

**Published:** 2025-11-20

**Authors:** Yanlang He, Lifen Liang, Sheng Wei

**Affiliations:** 1 Department of Infectious Disease, Shaoyang Central Hospital, Shaoyang, China; 2 Department of General Medicine, The Second Affiliated Hospital of Wannan Medical College, Wuhu, China

**Keywords:** neurobrucellosis, intracranial abscess, head trauma, atypical presentation, MRI

## Abstract

Giant intracranial *Brucella* abscess is a severe and rare central nervous system infection whose pathogenesis remains incompletely understood. We detail the case of a 75-years-old urban male without *Brucella* exposure history who presented with fever and headache. Initial attribution of cephalgia to head trauma delayed diagnosis and treatment. Magnetic resonance imaging, metagenomic next-generation sequencing, and cerebrospinal fluid culture confirmed rapid development of a giant *Brucella* abscess (31 mm × 58 mm) within 2 weeks after head trauma. Head trauma may be potentially associated with the formation of *Brucella* brain abscess. Consequently, brucellosis patients with recent head trauma may warrant vigilant monitoring for this rare complication. It is imperative to avoid the premature attribution of headache to head trauma in such patients, as such an oversight risks delaying the diagnosis and management of a *Brucella* brain abscess.

## Introduction

Brucellosis is a globally distributed zoonotic disease, with an estimated annual incidence of at least 1.6–2.1 million new cases of human brucellosis worldwide (1). It is commonly found among pastoralists exposed to infected animal secretions (e.g., during animal delivery assistance or slaughtering of cattle and sheep), while a smaller proportion of cases result from consuming unpasteurized dairy products (2). In China, the incidence of brucellosis has shown a persistent upward trend. Between 1950 and 2024, the incidence rate increased from 0.002/100,000 to 4.949/100,000. While the majority of human infections still occur among farmers and herders, the ongoing urbanization also places the urban population at risk of infection. Brucellosis not only causes significant economic losses in agriculture but also poses a substantial public health problem (3). The disease frequently involves multiple systems and organs in affected patients. Osteoarticular involvement represents the most prevalent complication, occurring in approximately 2%–77% of cases, typically manifesting as spondylitis, sacroiliitis, or peripheral arthritis (4).

However, neurobrucellosis (NB) is a rare complication, occurring in approximately 4% of brucellosis patients (5). Due to the absence of typical clinical features, diagnosis rates are only about 1.7% in adults and 0.8% in children (6), frequently leading to misdiagnosis and delayed diagnosis. Neurobrucellosis can impair health and quality of life in various severe forms. Central nervous system involvement may manifest as encephalitis, meningoencephalitis, cerebellar ataxia, myelitis, or cranial nerve involvement. In severe cases, subarachnoid hemorrhage, pseudotumor cerebri, confusion, and even life-threatening conditions may occur (7). Peripheral nerve complications include neuropathy/radiculopathy, Guillain-Barré syndrome, and poliomyelitis-like syndromes (8, 9). Consequently, the clinical manifestations of neurobrucellosis are highly variable and non-specific. The most common symptom is headache, with other clinical manifestations including agitation, muscle weakness, disorientation, behavioral abnormalities, seizures, urinary and fecal incontinence, and hearing loss (7). The lack of pathognomonic symptoms makes early diagnosis challenging.

Head trauma can also cause headache in patients. If important neurons or brain parenchyma are injured, the aforementioned non-specific symptoms may also appear. If a patient with neurobrucellosis coincidentally experiences head trauma, the underlying condition might be masked in the early stages, making differential diagnosis challenging. Herein, we report the first case of a *Brucella* brain abscess in an urban elderly male after head trauma. It is exceptionally rare for a giant *Brucella* brain abscess to develop rapidly following head trauma. We aim to report this phenomenon to enhance clinicians’ vigilance for intracranial infections in brucellosis patients with a history of head trauma, thereby preventing delays in diagnosis and treatment.

## Case report

**FIGURE 1:**
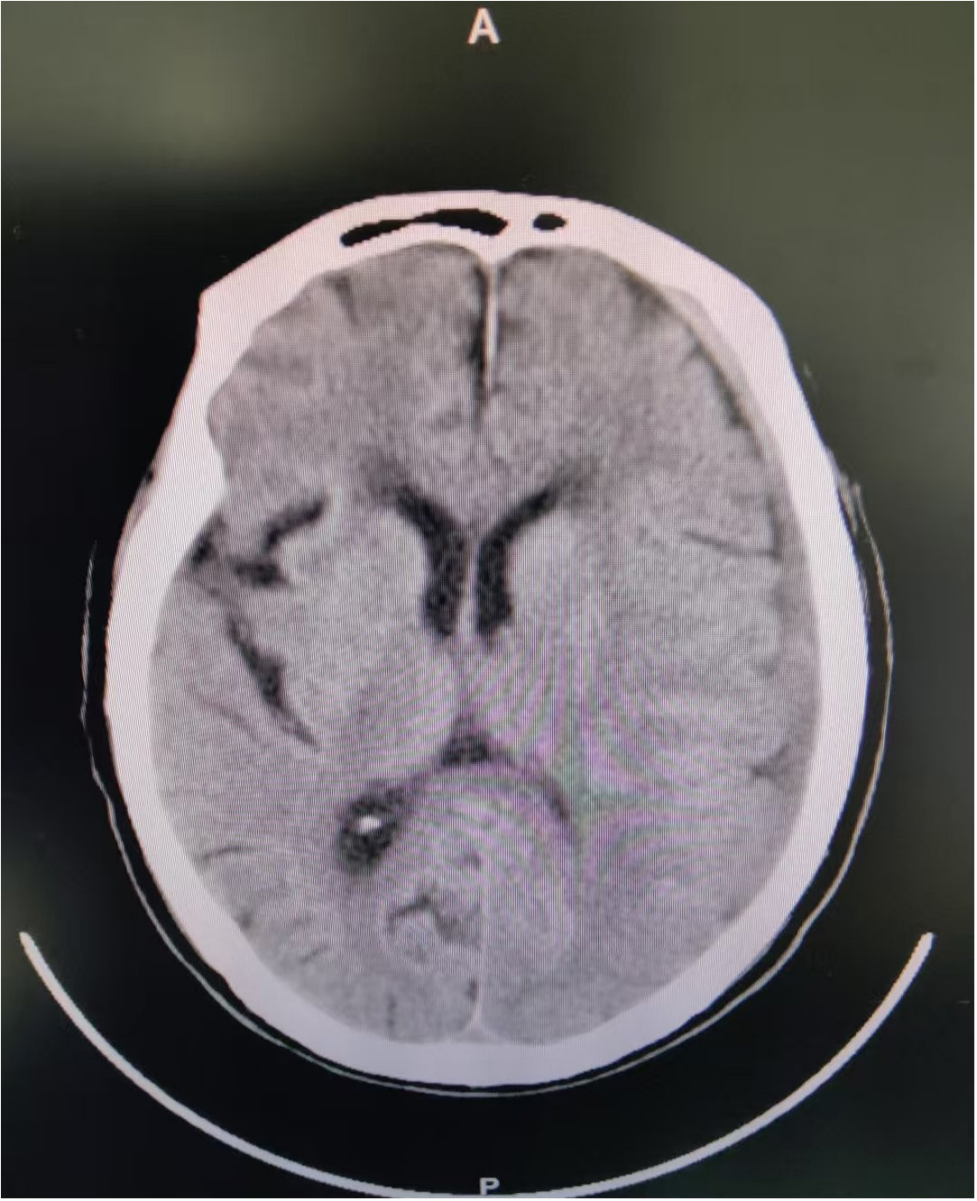
The patient’s brain CT examination after the car accident showed a small left subdural hematoma.

In April 2025, a 75-years-old male patient was admitted to the department of infectious diseases due to a 40-days history of fever. His past medical history was negative for tuberculosis, hematological disorders, or rheumatological diseases. He was a retired civil servant with no clinical background involving agricultural work, poultry contact, or consumption of unpasteurized meat or dairy products. Seven days prior to admission, he had been involved in a traffic accident, sustaining a mild impact to the frontal region of his head. After injury, he did not show any symptoms suggestive of a cerebrospinal fluid (CSF) leak, such as rhinorrhea or otorrhea. Since the impact to the head might have been mild, an initial head CT scan performed at a local hospital revealed only a minimal subdural hematoma without any radiological evidence of a skull base fracture (Figure 1), and consequently, no surgical intervention was undertaken.

On the day of admission, a detailed history was obtained. Apart from fever, he reported only intermittent headache, which had commenced following the traumatic injury. He denied nausea, vomiting, altered consciousness, seizures, or other manifestations suggestive of intracranial infection. Physical examination revealed no rash, subcutaneous hemorrhage, or lymphadenopathy. Hepatosplenomegaly was absent on palpation. Neurological examination, including assessment for pathological reflexes and meningeal signs, was negative. Apart from a fever of 38.8 °C, no other physical examination abnormalities were apparent. Laboratory investigations revealed a white blood cell (WBC) count of 12.21 × 10^9^/L with 90.5% neutrophils, and a procalcitonin (PCT) level of 5.7 ng/ml. Tests including purified protein derivative (PPD) skin test, Weil-Felix test, (1,3)-β-D-glucan assay (*G*-test), galactomannan assay (GM test), Epstein-Barr virus (EBV) DNA load, cytomegalovirus (CMV) DNA load, hepatitis B surface antigen, HIV antibody, and *Treponema pallidum* antibody were all negative. Bone marrow aspiration and lumbar puncture were not performed due to patient refusal. Echocardiography, chest computed tomography (CT), and abdominal CT scans showed no abnormalities.

However, despite the absence of an epidemiological history, brucellosis-specific serology returned positive results: *Brucella* IgG antibody was detected positive, the standard tube agglutination test (SAT) titer was 1:400, and the rose bengal plate agglutination test (RBT) was positive. Consequently, on hospital day 3, he was diagnosed with brucellosis. In accordance with the Chinese guidelines for brucellosis, treatment was initiated with doxycycline (0.1 g every 12 h) combined with rifampicin (0.6 g once daily).

Beginning on hospital day 4, the patient’s temperature normalized. However, he continued to report recurrent headaches. Attributing this to his traumatic history, we suspected the subdural hematoma was the cause. Neurosurgery consultation recommended symptomatic management with rotundine (60 mg every 12 h) for analgesia and atorvastatin to potentially promote hematoma resolution. Despite this, his headaches persisted without improvement; the duration of episodes increased, and the pain intensity progressively worsened.

**FIGURE 2:**
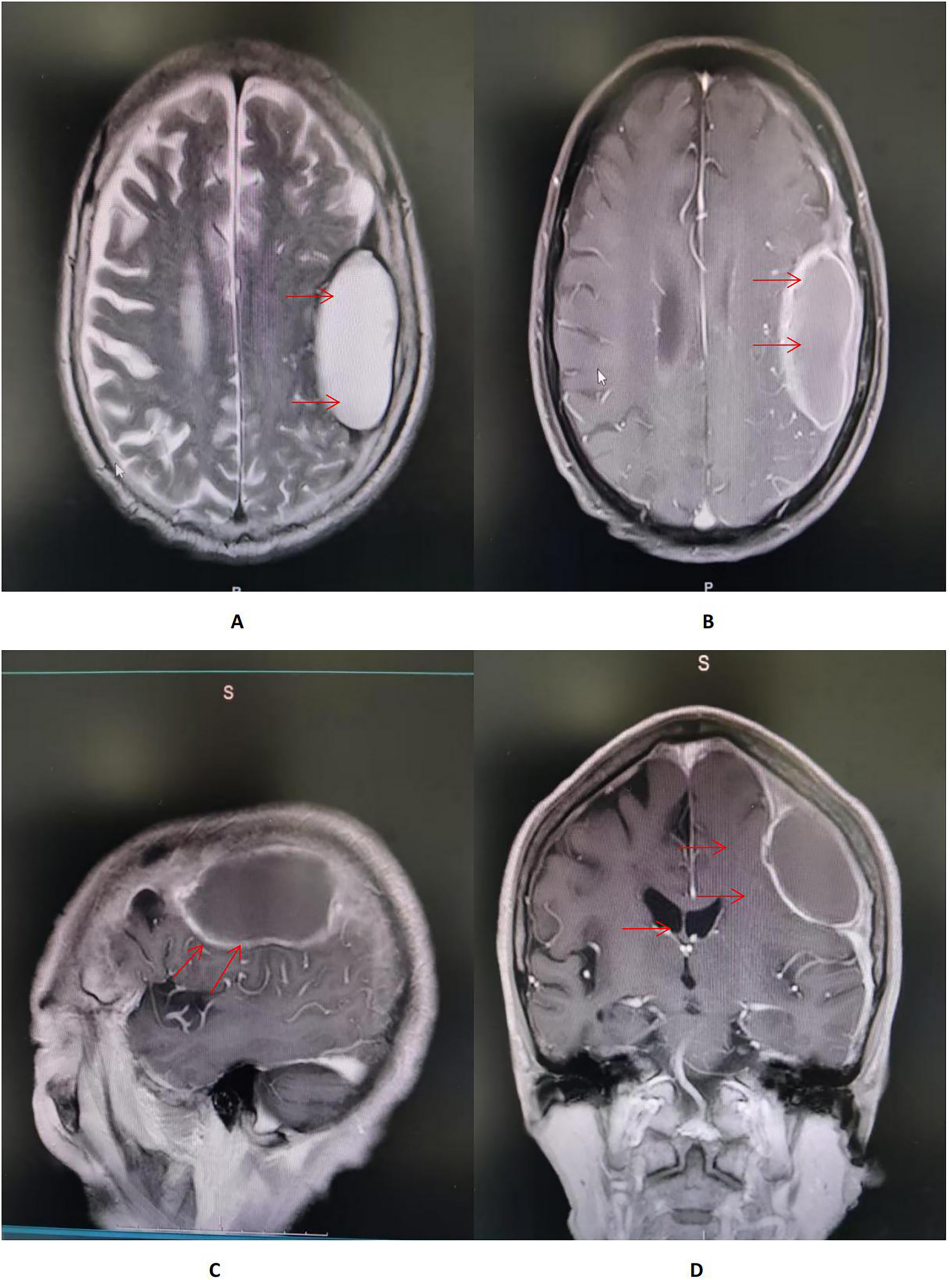
**(A,B)** Axial T2-weighted/FLAIR and T1-weighted MRI of the head demonstrate a mass-like abnormal signal focus (red arrow) in the left frontoparietal region, measuring approximately 31 × 58 mm. The lesion appeared hyperintense on T2-weighted/FLAIR images and iso- to hypointense on T1-weighted images, situated immediately beneath the inner table of the skull. A moderate perilesional edema was observed, with localized effacement of adjacent sulci. No significant midline shift was identified. **(C)** Sagittal contrast-enhanced T1-weighted MRI of the head showed an in homogeneously enhancing lesion in the left frontoparietal region, abutting the dura mater with a prominent “dural tail sign” (red arrow). The lesion had well-defined margins, and the surrounding edema showed no enhancement. **(D)** Coronal contrast-enhanced T1-weighted MRI of the head demonstrated a large mass lesion with rim enhancement and a non-enhancing central area, suggestive of cystic or necrotic components. A significant perilesional edema was present, causing mild mass effect and displacement of the surrounding brain structures (red arrow).

**FIGURE 3:**
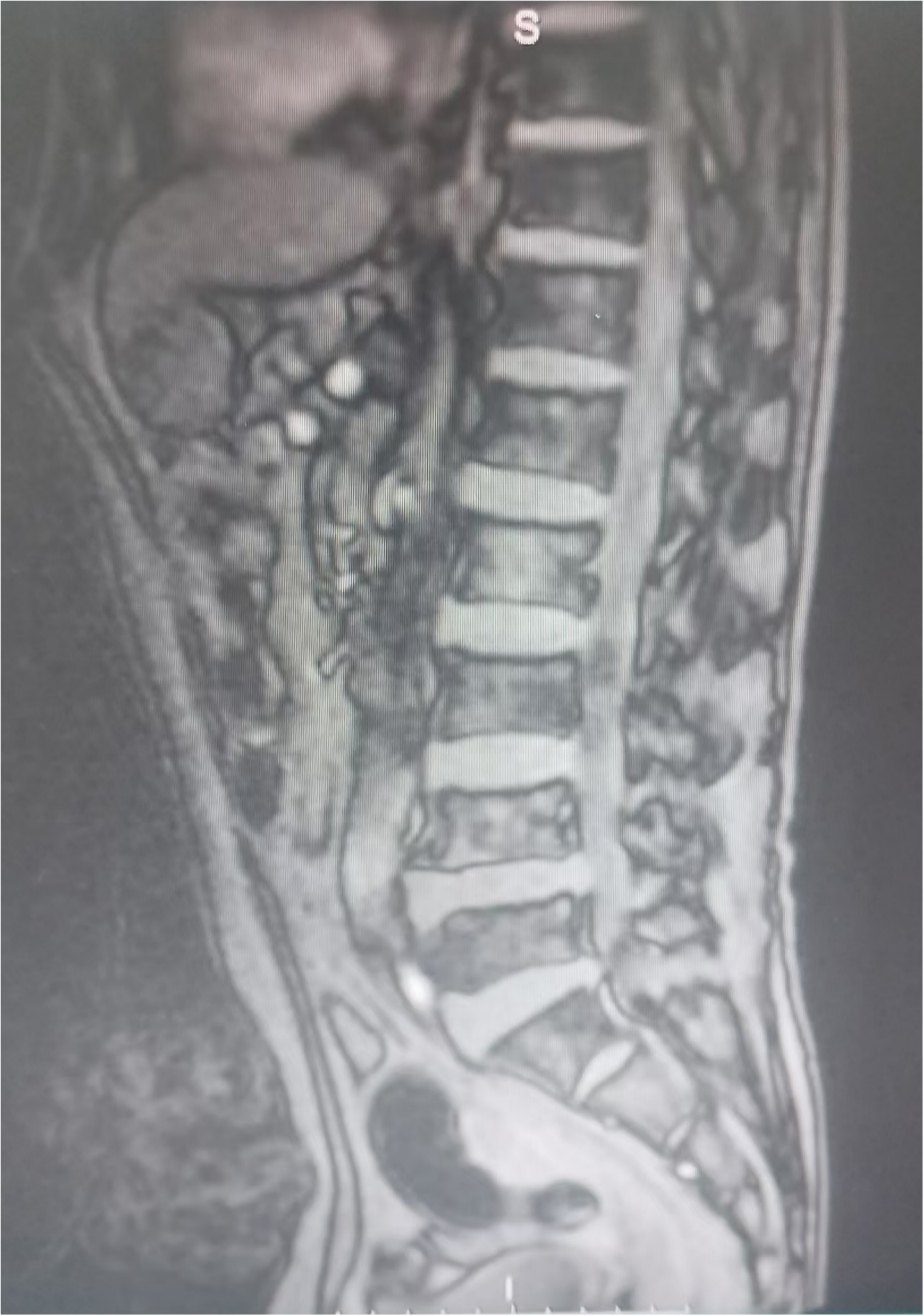
The patient’s spinal MRI showed no abnormalities.

On hospital day 7, axial T2-weighted/FLAIR and T1-weighted MRI of the head demonstrate a mass-like abnormal signal focus in the left frontoparietal region, measuring approximately 31 mm × 58 mm. The lesion appeared hyperintense on T2-weighted/FLAIR images and iso- to hypointense on T1-weighted images. A significant perilesional edema was present in coronal contrast-enhanced T1-weighted MRI, causing mild mass effect and displacement of the surrounding brain structures (Figure 2). Spinal MRI showed no abnormal signal intensity suggestive of involvement (Figure 3). Lumbar puncture was immediately performed. Opening pressure was elevated at 220 mmH2O, and the CSF appeared turbid. Routine CSF analysis (Table 1) demonstrated: white blood cell (WBC) count 460/mm^3^, Pandy’s test positive, total protein 1312 mg/L, glucose 0.99 mmol/L, chloride 115.9 mmol/L, and a standard tube agglutination test (SAT) titer of 1:480. Metagenomic next-generation sequencing (mNGS) of the CSF detected 3489 sequence reads of *Brucella melitensis*. The diagnosis was revised to neurobrucellosis (meningitis) complicated by giant intracranial abscess formation. Simultaneously, we cultured the cerebrospinal fluid on Columbia blood agar plates at an ambient temperature of 35 degrees Celsius. Following the current Chinese guidelines for neurobrucellosis (10), antimicrobial therapy was intensified with the addition of ceftriaxone (2 g every 12 h). Mannitol (125 mL every 8 h) was initiated for intracranial pressure reduction, and dexamethasone (10 mg once daily) was added to mitigate inflammatory exudation. Subsequently, the patient’s headaches began to subside.

**TABLE 1:** Results of serial cerebrospinal fluid (CSF) examinations.

Parameter	Initial CSF examination	Follow-up CSF examination	Reference range	Unit
Appearance	Cloudy	Clear and colorless	Clear, colorless	–
White blood cell (WBC) count	460	10	–	/mm∧3
Pandy’s test (globulin)	Positive	Positive	Negative	–
Coagulum	Absent	Absent	–	–
India ink preparation (for *Cryptococcus neoformans*)	Negative	Negative	Negative	–
Mononuclear cells	32	–	–	%
Polymorphonuclear cells	68	–	–	%
Adenosine deaminase (ADA)	4.1	1	0–8	U/L
Total protein	1312.1	956.1	150–450	mg/L
Glucose	0.99	3.46	2.5–4.4	mmol/L
Chloride	115.9	127.4	120–130	mmol/L
IgG antibody (ELISA)	Positive	Positive	Negative	–
Tube agglutination test titer	1:480	1:320	–	–
Rose bengal plate agglutination test (RBT)	Positive	Positive	Negative	–

**FIGURE 4:**
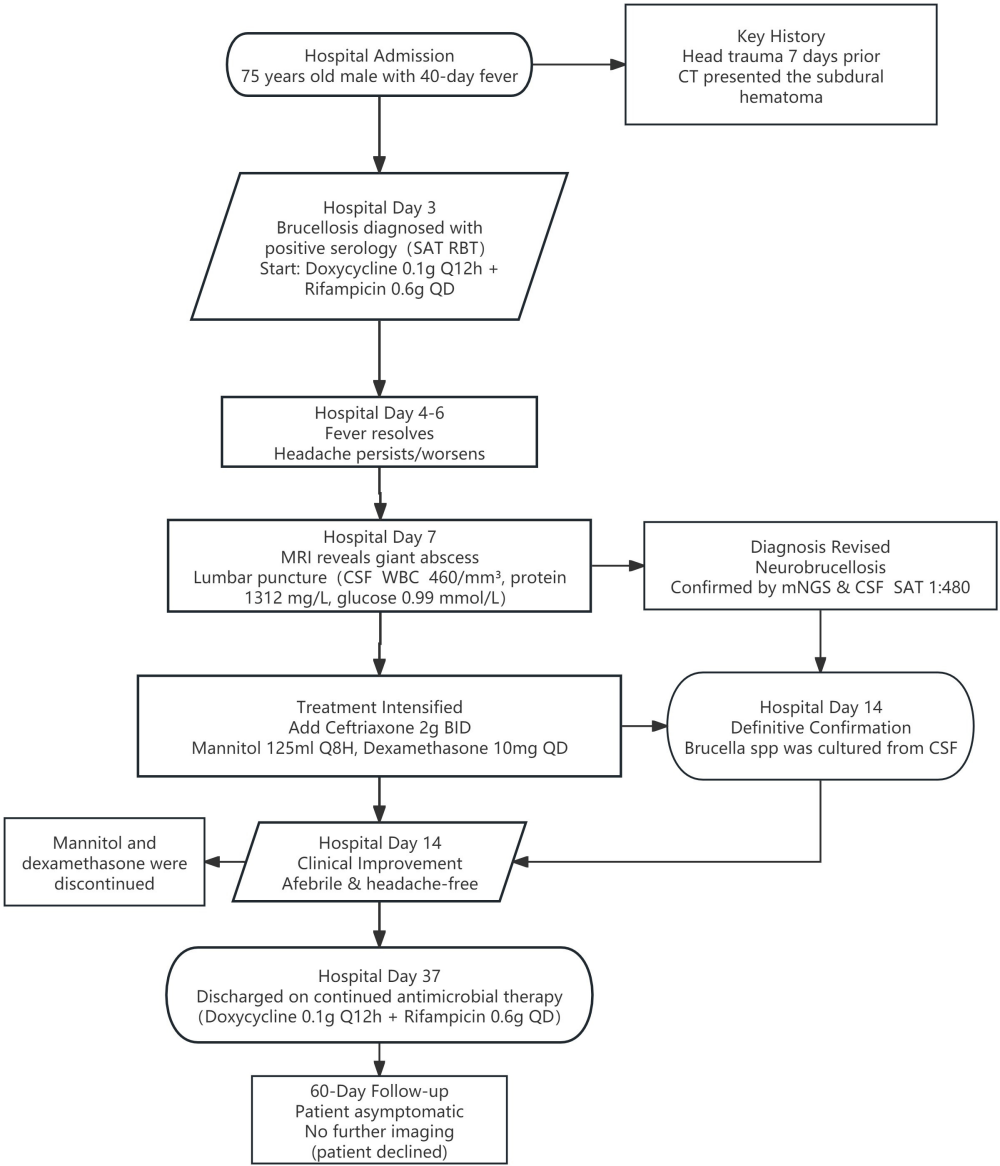
Diagnostic and treatment timeline of this patient with neurobrucellosis.

On hospital day 14, the laboratory technician performed a slide agglutination test on small, round, smooth, transparent suspicious colonies. Visible agglutination occurred within 2 min, identifying the colonies as *Brucella* spp., definitively confirming our diagnosis. At this point, the patient’s headache had resolved, and we discontinued dexamethasone and mannitol. By hospital day 20, the patient remained afebrile and free of headache. Follow-up CSF analysis demonstrated a decline in brucellosis-specific antibody titers (Table 1). The patient was discharged home on hospital day 37 to continue antimicrobial therapy. Based on the recommendations of the Chinese expert consensus on the diagnosis and treatment of neurobrucellosis (10), we discontinued ceftriaxone and advised the patient to continue oral doxycycline (0.1 g every 12 h) combined with rifampicin (0.6 g once daily) for a total course of 6 months. At the 60-days post-discharge follow-up, he remained asymptomatic. Repeat cranial MRI and CSF examination were not performed as the patient declined further investigations. Figure 4 showed the process of case presentation and follow up.

## Discussion

Neurobrucellosis complicated by giant intracranial abscess represents an exceptionally rare event globally. A systematic literature review (Table 2) indicates that since 1980, only 13 cases of brucellosis-associated intracranial abscess have been reported (11–23). Fewer than five of these involved abscesses exceeding 5 cm in diameter. Notably, the majority of documented cases occurred in children, and most patients had identifiable epidemiological exposure histories. To the best of our knowledge, this is the first reported case of *Brucella meningitis* complicated by giant *Brucella* abscess formation in an urban-dwelling elderly male patient without established epidemiological exposure.

**TABLE 2:** Literature review of brucellosis-associated intracranial abscesses (1980–2025).

Year	Age	Sex	Epidemiological exposure history	Abscess characteristics	Treatment regimen	Outcome	Reference
1990	12	M	Family engaged in farming and animal breeding	5 × 6 × 7 cm	Doxycycline (120 mg/day), rifampicin (480 mg/day), ampicillin-sulbactam (8 g/day)	Cured	Kalelioğlu M, Ceylan S, Köksal I, Kuzeyli K, Aktürk F. Brain abscess caused by *Brucella abortus* and *Staphylococcus aureus* in a child. Infection. 1990;18(6):386-387. doi: 10.1007/BF01646416 (11)
2006	70	M	Not specified	Not specified	Doxycycline (100 mg BID), rifampicin (300 mg BID)	Cured	Koc K. Brucellar brain abscess and bilateral arachnoid cysts, unilaterally complicated by subdural hematoma. J Clin Neurosci. 2006;13(4):485-487. doi: 10.1016/j.jocn.2005.06.012 (12)
2016	8	F	Rural residence; father (farmer); sister with brucellosis	50 mm × 40 mm	Ceftriaxone, rifampicin, trimethoprim-sulfamethoxazole	Cured	Yilmaz S, Avcu G, Beyazal M, Arslan M. A rare cause of seizures: brucellar brain abscess. Braz J Infect Dis. 2016;20(3):310-311. doi: 10.1016/j.bjid.2015.12.010 (13)
2023	55	M	Consumption of unpasteurized dairy products	8 mm × 8 mm (pituitary)	Doxycycline, rifampicin, ceftriaxone	Cured	De la Peña-Sosa G, Cabello-Hernández AI, Gómez-Ruíz RP, Gómez-Sámano MA, Gómez-Pérez FJ. Pituitary Abscess Causing Panhypopituitarism in a Patient With Neurobrucellosis: Case Report. AACE Clin Case Rep. 2023;10(1):10-13. Published 2023 Oct 29. doi: 10.1016/j.aace.2023.10.005 (14)
1999	30	F	Not specified	Pituitary abscess (size unspecified)	Trimethoprim-sulfamethoxazole, rifampicin	Cured	Güven M.B., Cirak B., Kutluhan A., Ugras S. Pituitary abscess secondary to neurobrucellosis. Case illustration. J Neurosurg. 1999;90(6):1142. doi: 10.3171/JNS.1999.90.6.1142 (15)
2018	25	M	Not specified	Pontine abscess (size unspecified)	Not specified	Cured	Turkoglu SA, Halicioglu S, Sirmatel F, Yildiz M, Yildiz N, Yildiz S. Vasculitis and neurobrucellosis: Evaluation of nine cases using radiologic findings. Brain Behav. 2018;8(4):e00947. Published 2018 Mar 9. doi: 10.1002/brb3.947 (16)
1989	4	M	Not specified	6 multifocal abscesses (2 × 2 to 4 cm × 4 cm)	Streptomycin, tetracycline	Cured	Guvenc H, Kocabay K, Okten A, Bektas S. Brucellosis in a child complicated with multiple brain abscesses. Scand J Infect Dis. 1989;21(3):333-336. doi: 10.3109/00365548909035705 (17)
2004	70	M	Regular consumption of unpasteurized milk/cheese	Multiloculated lesion (right occipital lobe; size unspecified)	Doxycycline (100 mg BID), rifampicin (300 mg BID)	Cured	Gündeş S, Meriç M, Willke A, Erdenliğ S, Koç K. A case of intracranial abscess due to *Brucella melitensis*. Int J Infect Dis. 2004;8(6):379-381. doi: 10.1016/j.ijid.2004.05.003 (18)
2000	60	F	Occupation: farming	Chronic optic chiasm abscess	Doxycycline (100 mg BID), rifampicin (300 mg BID)	Cured	Stranjalis G, Singounas E, Boutsikakis I, Saroglou G. Chronic intracerebral *Brucella* abscess. Case illustration. J Neurosurg. 2000;92(1):189. doi: 10.3171/jns.2000.92.1.0189 (19)
1993	3	M	Consumption of raw milk	Right cerebellar abscess (size unspecified)	Rifampicin (20 mg/kg/day), trimethoprim-sulfamethoxazole (10 mg/kg/day)	Cured	al-Eissa YA. Unusual suppurative complications of brucellosis in children. Acta Paediatr. 1993;82(11):987-992. doi: 10.1111/j.1651-2227.1993.tb12617.x (20)
2005	30	M	Not specified	Right inferior cerebellar peduncle (size unspecified)	Doxycycline (100 mg BID), rifampicin (300 mg BID), ceftriaxone	Cured	Kizilkilic O, Turunc T, Yildirim T, Demiroglu YZ, Hurcan C, Uncu H. Successful medical treatment of intracranial abscess caused by *Brucella* spp. J Infect. 2005;51(1):77-80. doi: 10.1016/j.jinf.2004.08.021 (21)
2017	55	M	Consumption of unpasteurized dairy products	14 cm × 4 cm	Ceftriaxone (2 g BID), doxycycline (100 mg BID), rifapentine (700 mg/day)	Cured	Zhang J, Chen Z, Xie L, et al. Treatment of a subdural empyema complicated by intracerebral abscess due to *Brucella* infection. Braz J Med Biol Res. 2017;50(5):e5712. Published 2017 Mar 30. doi: 10.1590/1414-431 × 20165712 (22)
2006	12	M	Consumption of unpasteurized dairy products	Multiple small abscesses (peripontine/brainstem/ cerebellum)	Rifampicin (15 mg/kg/day), trimethoprim-sulfamethoxazole (10 mg/kg/day)	Cured	Keihani-Douste Z, Daneshjou K, Ghasemi M. A quadriplegic child with multiple brain abscesses: case report of neurobrucellosis. Med Sci Monit. 2006;12(12):CS119-CS122. (23)

F stands for female and M for male. BID means twice daily.

First, the absence of a clear epidemiological history in our reported case is a distinctive highlight. Epidemiological studies indicate that individuals at the highest risk for *Brucella* infection include veterinarians, artificial insemination service personnel, zoo technicians, ranch workers, and employees in meat processing plants. However, this patient was an urban retiree with no contact with animal husbandry, making his acquisition of *Brucella* infection seemingly unusual and puzzling. In reality, *Brucella* can be transmitted to humans through various routes. While the most common modes involve direct contact with infected animal secretions (e.g., during assisted animal delivery or slaughter of cattle and sheep) or consumption of unpasteurized dairy products, we should not overlook the less common transmission routes. These include direct contact of wounds or mucous membranes with surfaces or objects contaminated with *Brucella*, and inhalation of infectious aerosols (24). Furthermore, rare cases of transmission via sexual contact, blood transfusion, or vertical mother-to-child transmission have been documented (25, 26). It is possible that this patient was infected through one of these less common routes, which could not be definitively identified, potentially due to recall bias. This case cautions against underestimating the transmission potential of routes previously considered uncommon. Simultaneously, this case suggests that diagnostic reasoning for *Brucella* infection in clinical practice should not be entirely constrained by epidemiological history. Maintaining a high index of suspicion for this disease, even in non-endemic areas or among individuals without high-risk occupations, is crucial to avoid missed diagnoses.

Neurobrucellosis represents a severe yet uncommon complication of human brucellosis, associated with considerable mortality rates as high as 7% if accurate diagnosis is missed (27). Therefore, prevention and early diagnosis are paramount for reducing mortality and improving prognosis. Based on the transmission routes mentioned above, public prevention should combine source control and protection of susceptible populations. Firstly, the WHO recommends strict implementation of animal vaccination and quarantine in pastoral areas, along with enhanced safety supervision of animal-derived food production, to control the source of transmission (28). Secondly, occupational exposure is a significant issue in *Brucella* transmission, and professionals in specific fields should adopt measures to strengthen occupational protection (24). Finally, public health education is also a vital method for preventing human *Brucella* infection (29, 30). Additionally, based on the present case, the rapid development of a *Brucella* brain abscess following head trauma suggests that avoiding head injury might be one of the preventive measures against neurobrucellosis.

On the other hand, early diagnosis can be challenging in some patients due to the absence of characteristic signs and symptoms. Literature indicates that the diagnosis of neurobrucellosis is based on four criteria: signs and symptoms suggestive of neurobrucellosis, cerebrospinal fluid (CSF) findings consistent with neurobrucellosis, positive identification of *Brucella* spp. in the CSF and/or the presence of antibodies against *Brucella* in the CSF, and supportive diagnostic imaging (such as cranial magnetic resonance imaging). Specifically, patients with neurobrucellosis may present with fever, headache, neck stiffness, cranial nerve palsies, aphasia, psychiatric symptoms, confusion, vomiting, ataxia, and seizures (31). However, the clinical features of giant *Brucella* brain abscess in the elderly population remain poorly characterized. Our case showed that headache may sometimes be the sole prominent manifestation of *Brucella meningitis* complicated by giant intracranial abscess, which was consistent with previously reported pediatric cases of *Brucella* brain abscess (13, 17). Based on our case, we recommend that clinicians maintain a high index of suspicion for intracranial infection in brucellosis patients with a history of head trauma. Headaches should not be attributed solely to the trauma.

Moreover, the characteristic CSF biochemical profile in neurobrucellosis often shows protein levels typically >45 mg/dL, a CSF/serum glucose ratio usually <0.4, and mild pleocytosis predominantly with lymphocytes, findings that closely resemble those of tuberculous, syphilitic, and viral nervous system infections (32, 33). Our case results were largely consistent with this profile, aligning with previous research which found no evidence of significant differences in CSF biochemical test results among patients with different clinical phenotypes of neurobrucellosis (34).

The most critical investigations for diagnosing neurobrucellosis are CSF microbiological studies and imaging. Although CSF culture positive for *Brucella* is the gold standard, its yield is positive in less than 25% of cases and it is time-consuming (35). Therefore, immunological tests and molecular techniques have become common adjunctive diagnostic tools, especially in culture-negative cases. The CSF standard tube agglutination (STA) test is a highly sensitive and specific serological test for detecting *Brucella* antibodies and can thus be used to diagnose neurobrucellosis. Although two studies have reported a CSF titer of ≥1:80 as indicative of positive antibodies (36, 37), a consensus has not been reached, and many studies still report any positive titer. However, it is important to note that patients with only a low CSF SAT titer and no other diagnostic criteria should not be classified as having neurobrucellosis, as peripherally produced *Brucella* antibodies might minimally cross the compromised blood-brain barrier into the CSF. Furthermore, in recent years, metagenomic next-generation sequencing (mNGS) of CSF has been increasingly applied for diagnosing neurobrucellosis due to its higher sensitivity. A study from Northwestern China, an endemic area for brucellosis, found that from 2015 to 2021, the sensitivity of mNGS for detecting *Brucella* in CSF was 90%, compared to 54.5% for CSF culture (38). Consequently, there is a strong suggestion to utilize mNGS for diagnosing neurobrucellosis, particularly in non-endemic areas (5). In our case, positive results were obtained from CSF culture, SAT, and mNGS, further supporting the clinical value of these tests in neurobrucellosis.

Imaging plays a significant role in the diagnosis and differential diagnosis of neurobrucellosis. Neurobrucellosis can present with four types of imaging findings: normal appearance, inflammatory changes characterized by abnormal enhancement, white matter changes, and vascular changes. Inflammatory changes can manifest as either diffuse, primarily leptomeningeal enhancement, or focal inflammatory lesions, including encephalitis/myelitis, nerve root enhancement, granulomas, and abscess formation (39). However, imaging features of *Brucella* brain abscesses are rarely reported, and whether they possess specific inflammatory changes remains inconclusive. Our case provides a reference: MRI showed meningeal thickening, a ring-enhancing lesion with no enhancement in the central necrotic area of the abscess, and surrounding edema of varying degrees. Its imaging appearance is similar to brain abscesses caused by other bacteria, necessitating thorough correlation with microbiological results for diagnosis.

In the present case, the initial CT scan failed to detect the abscess when head trauma occurred. However, a giant *Brucella* abscess was evident on MRI performed just 2 weeks following the head trauma. This temporal sequence suggests that the head trauma may be potentially associated with the occurrence of the *Brucella* brain abscess. This may represent a novel contributing factor not previously highlighted in the literature concerning Brucellar abscess formation, but the precise underlying mechanisms still warrant further elucidation. The absence of skull base fracture or cerebrospinal fluid leakage led us to consider the direct intracranial invasion of *Brucella* bacteria via the respiratory tract and skull base as a less likely pathway for brain abscess formation. Existing literature posits that *Brucella* spp. invades the reticuloendothelial system, causing bacteremia. Subsequently, it breaches the blood-brain barrier (BBB), leading to neurobrucellosis via mechanisms including the release of harmful cytokines or endotoxins, direct neuropathological effects, and host inflammatory and immune responses (40). However, in clinical practice, neurobrucellosis remains an uncommon manifestation, largely attributable to the inherent difficulty *Brucella* faces in penetrating the intact BBB. Based on our case and previous literature, it may represent a potential direction for future research to investigate the complex relationship between head trauma, blood-brain barrier disruption, and the intracranial invasion and colonization of *Brucella*.

It must be acknowledged that this case report has several limitations. Firstly, the conclusions are based on a single case report, and the supporting evidence is relatively limited, which restricts its generalizability. Secondly, the patient’s advanced age may have introduced recall bias regarding the epidemiological history. Third, the patient’s initial refusal of lumbar puncture led to a delay in diagnosis. Early CSF analysis is essential for the timely diagnosis of patients with unexplained persistent fever and headache, and lumbar puncture should be performed as early as possible. Furthermore, the patient declined follow-up imaging investigations, which impaired our final assessment regarding the route of infection and treatment outcome. Finally, the mechanism underlying the rapid development of the *Brucella* brain abscess following head trauma remains unclear; we have merely proposed a hypothesis that requires cautious interpretation and further investigation.

## Conclusion

In patients with brucellosis and a recent history of head trauma, clinicians should remain alert to the possibility of *Brucella* brain abscess–a rare complication. Headaches should not be automatically attributed to the trauma alone, as this assumption may lead to delayed diagnosis or even missed identification of this serious infection.

## Data Availability

The original contributions presented in this study are included in this article/supplementary material, further inquiries can be directed to the corresponding author.
